# Genome-Wide Analysis of Cell-Free DNA Methylation Profiling for the Early Diagnosis of Pancreatic Cancer

**DOI:** 10.3389/fgene.2020.596078

**Published:** 2020-12-02

**Authors:** Shengyue Li, Lei Wang, Qiang Zhao, Zhihao Wang, Shuxian Lu, Yani Kang, Gang Jin, Jing Tian

**Affiliations:** ^1^Key laboratory of Resource Biology and Biotechnology in Western China, Ministry of Education, School of Medicine, Northwest University, Xi’an, China; ^2^Department of Gastroenterology, Changhai Hospital, Second Military Medical University, Shanghai, China; ^3^School of Biomedical Engineering, Bio-ID Center, Shanghai Jiao Tong University, Shanghai, China; ^4^Department of General Surgery, Changhai Hospital, Second Military Medical University, Shanghai, China

**Keywords:** pancreatic ductal adenocarcinoma, cfDNA, MeDIP-seq, methylation, biomarkers

## Abstract

As one of the most malicious cancers, pancreatic cancer is difficult to treat due to the lack of effective early diagnosis. Therefore, it is urgent to find reliable diagnostic and predictive markers for the early detection of pancreatic cancer. In recent years, the detection of circulating cell-free DNA (cfDNA) methylation in plasma has attracted global attention for non-invasive and early cancer diagnosis. Here, we carried out a genome-wide cfDNA methylation profiling study of pancreatic ductal adenocarcinoma (PDAC) patients by methylated DNA immunoprecipitation coupled with high-throughput sequencing (MeDIP-seq). Compared with healthy individuals, 775 differentially methylated regions (DMRs) located in promoter regions were identified in PDAC patients with 761 hypermethylated and 14 hypomethylated regions; meanwhile, 761 DMRs in CpG islands (CGIs) were identified in PDAC patients with 734 hypermethylated and 27 hypomethylated regions (*p*-value < 0.0001). Then, 143 hypermethylated DMRs were further selected which were located in promoter regions and completely overlapped with CGIs. After performing the least absolute shrinkage and selection operator (LASSO) method, a total of eight markers were found to fairly distinguish PDAC patients from healthy individuals, including *TRIM73*, *FAM150A*, *EPB41L3*, *SIX3*, *MIR663*, *MAPT*, *LOC100128977*, and *LOC100130148*. In conclusion, this work identified a set of eight differentially methylated markers that may be potentially applied in non-invasive diagnosis of pancreatic cancer.

## Introduction

Pancreatic ductal adenocarcinoma (PDAC) is one of the most highly aggressive diseases in the world. Due to the hard challenge of detecting the disease at an early stage, poor prognosis often occurs. The morbidity of PDAC is approximately close to that of mortality. Nearly 80% of PDAC patients have no early symptoms before the advanced stage (Kaur et al., 2012) with a 5-year survival rate as low as 9% (Siegel et al., 2019). Accordingly, PDAC is the fourth leading cause of cancer-related death worldwide and is predicted to rise to second place by 2030 (Rahib et al., 2014). Currently, ultrasonography, computed tomography, positron emission tomography, magnetic resonance imaging, and endoscopic ultrasonography are the most commonly used diagnostic methods for PDAC (Kamisawa et al., 2016; Chu et al., 2017). However, operator experience, patient obesity and intestinal gas, and other factors affect the accuracy of diagnosis (Kamisawa et al., 2016). In addition, due to the location of the pancreas, it is not easy to make an early diagnosis compared to other digestive tract tumors (Lowenfels and Maisonneuve, 2004). Therefore, it would be very valuable to identify both sensitive and specific non-invasive biomarkers for the early diagnosis of PDAC.

Epigenetic regulation, especially DNA methylation, plays an important role in the regulation of gene expression and the development of cancers. Genome-wide hypomethylation is common in cancer cells, leading to genomic instability. Some tumor suppressor genes with promoter hypermethylation are observed to cause gene silencing (Hanahan and Weinberg, 2000; Esteller, 2007). Hypermethylation of CpG islands (CGIs) in the promoters of tumor suppressor genes is a major and early event during tumorigenesis (Hanahan and Weinberg, 2000; Park et al., 2011; Udensi and Tchounwou, 2016; Liu et al., 2019). Aberrant methylation of promoter CGI regions in some genes has been proven to be associated with tumorigenesis and tumor growth (Cai et al., 2011; Pistore et al., 2017). Therefore, it is vital to detect the hypermethylation of promoter CpG islands for early diagnosis. This may contribute to the early detection of cancer and improve the therapeutic effect.

In recent years, circulating cell-free DNA (cfDNA), known as liquid biopsy, has attracted much more attention from the medical community due to its clinical advantages. As small double-stranded DNA fragments, cfDNA is released by necrotic or apoptotic cells and is circulated in the peripheral blood (Jahr et al., 2001; Stroun et al., 2001). During tumorigenesis, the increase of cell necrosis and apoptosis leads to the accumulation of cfDNA, which can be detected at a relatively early stage. Furthermore, cfDNA not only contains the same mutations as tumor cells, but also has the same methylation pattern, making it possible and convenient for early cancer diagnosis, even for those hidden organs such as the pancreas and bile ducts (Schwarzenbach et al., 2011).

Methylated DNA immunoprecipitation coupled with high-throughput sequencing (MeDIP-seq) is a sensitive technology for the detection of DNA methylation, which can even detect an initial DNA amount as low as 1 ng (Taiwo et al., 2012; Zhao et al., 2014). Genome-wide detection of cfDNA methylation profiling using the MeDIP-seq method has been developed recently for screening potential biomarkers of cancers in early stages. Based on cfDNA methylation patterns by MeDIP-seq analysis, (Shen et al., 2018) identified different potential biomarkers in pancreatic ductal adenocarcinoma, colorectal cancer, breast cancer, lung cancer, renal cancer, bladder cancer, and acute myeloid leukemia for early-stage detection. Xu et al. (2019) also identified a set of potential biomarkers that could be served in lung cancer clinical diagnosis by screening cfDNA methylation profiling using MeDIP-seq.

Therefore, in this study, we aimed to investigate the potential cfDNA methylation biomarkers in the diagnosis of PDAC. By MeDIP-seq analysis, we compared the differentially methylated regions (DMRs) of PDAC cfDNA with that of normal control, and identified 143 hypermethylated DMRs which were located in promoter regions and completely overlapped with CGIs in PDAC patients. After cross-validation with publicly available DNA methylation data, including 339 pancreatic adenocarcinoma (PAAD) patients and 357 normal controls, we successfully identified eight probes from six differentially methylated genes, containing *TRIM73*, *FAM150A*, *EPB41L3*, *SIX3*, *MIR663*, *MAPT*, *LOC100128977*, and *LOC100130148*, which could be used as potential biomarkers for early detection for PDAC patients.

## Materials and Methods

### Sample Collection

A total of six samples including four PDAC patients and two healthy controls were used for this study. Four serum samples from PDAC patients were supplied by ChangHai Hospital. All of them signed informed consent forms. Specimens were collected and analyzed with the approval of the ethics committees of ChangHai Hospital and School of Medicine, Northwest University, respectively.

### cfDNA Extraction

First, 5 ml peripheral blood was collected using EDTA anticoagulant tubes before surgery and drug treatment. The plasma was purified by centrifuge for 15 min at 1500 × *g* within 6 h of collection. cfDNA was extracted from 800 μl aliquots of plasma using a QIAamp Circulating Nucleic Acid Kit (Qiagen, 55114) according to manufacturer’s protocol and quantified with Bioanalyzer 2100 (Agilent Technologies).

### MeDIP-seq Library Construction and Sequencing

The cfDNA MeDIP-seq library was prepared as we described previously (Xu et al., 2019). In short, approximately 20 ng cfDNA was ligated with Illumina barcode adapters using a KAPA Hyper Prep Kit (KAPA, KK8502). The constructed cfDNA libraries were denatured at 95°C for 10 min. The methylated cfDNA was separated from the cfDNA libraries by immunoprecipitation using the 5-Methylcytosine (5mC) Monoclonal Antibody (Epigentek, A-1014). MeDIP DNA was further amplified using a Q5 High-Fidelity DNA Polymerase (NEB, M0491). After quality assessment using Bioanalyzer 2100 (Agilent Technologies), amplified libraries were subjected to deep sequencing by the Illumina HiSeq 2000 platform.

### Data Processing and Analysis

MeDIP-seq raw data were processed using the Trimmomatic software (version 0.38) to filter out low-quality reads and Illumina adapters. The clean reads were mapped to the human reference genome GRCh37/hg19 (UCSC) using the Bowtie software (version 2.3.3.1) (Langmead et al., 2009). The differentially methylated regions (DMRs) between pancreatic cancer patients and healthy controls were calculated with the R package MEDIPS (version 1.36.0) (Lienhard et al., 2014), the coupling factor for CpG density was generated based on the normalization of the patient MeDIP-seq data. The function of region of interest (ROI) analysis in the MEDIPS package was specifically used to investigate the DNA methylation levels in UCSC CpG islands, CpG shore (∼2 Kb from islands), and CpG shelf regions (∼4 Kb from islands)^1^. Mapping results were visualized using Integrative Genomics Viewer (IGV) (Thorvaldsdottir et al., 2013). Pathway analysis was carried with the Ingenuity Pathway Analysis (IPA) software (Qiagen).

Illumina Infinium HumanMethylation 450K BeadChip Array (HM450K) data from The Cancer Genome Atlas (TCGA) project and Gene Expression Omnibus (GEO) were used to validate our MeDIP-seq results. A total of 696 HM450K sample sets including 339 PAAD patients and 357 normal controls were assembled from the TCGA^2^ and GEO (GSE49149 and GSE40279) databases. The information about the patient age and gender of 696 HM450K sample sets are supplied in Supplementary Table 1. The bioinformatics pipeline and R codes are available as supplementary code in zenodo^3^. The variable selection was performed using the LASSO method (Xu et al., 2017). We subsampled 75% of the dataset for model building. After 500 iterations, we selected the probes that appeared more than 450 times as covariates, and obtained a total of eight probes. We fitted a logistic regression model with these candidate markers and measured the classification performance of the binary classifier using an area under the ROC curve (AUC).

The Paired Student’s *t*-test was performed using the processed beta (β) values (proportion of the methylated signal over the total signal) to compare the DNA methylation levels in the probe regions between 339 PAAD sample and 357 normal samples, the *p*-value for each maker was corrected by multiple testing with the Benjamini-Hochberg procedure (Benjamini and Yekutieli, 2001).

Multivariate Cox regression analysis was performed to construct the prognostic model based on the AIC value. Kaplan-Meier curves were generated and used to perform survival analysis using GEPIA^4^.

## Results

### Analysis of Global cfDNA Methylation Profiling in Pancreatic Cancer by MeDIP-seq

**FIGURE 1:**
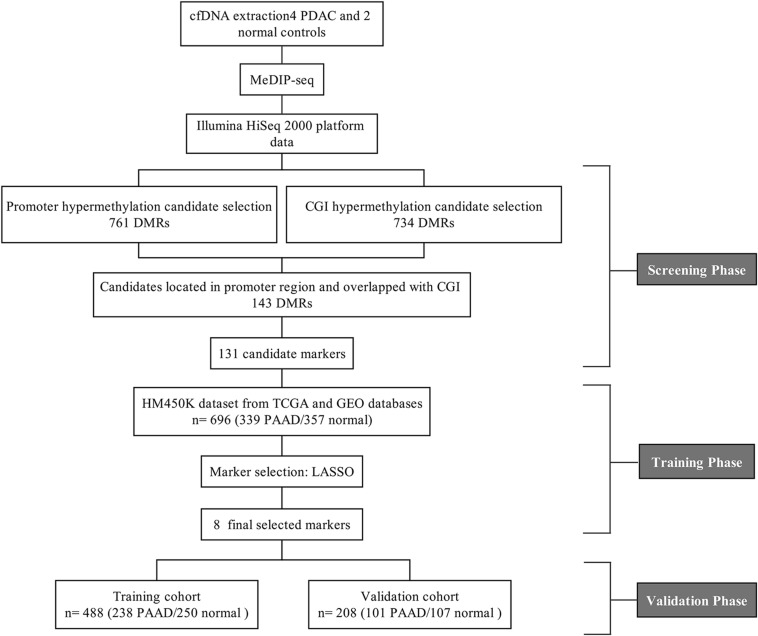
The flow chart of screening cfDNA methylation biomarkers in pancreatic cancer.

Four plasma samples of PDAC patients and two of healthy controls were collected, the clinical information of patients is shown in Table 1. The four PDAC samples were in the IB or IIB stage which had entered into the early or middle stage of pancreatic cancer (Table 1) (van Roessel et al., 2018). After being subjected to quality testing, the size of the cfDNA fragments was mainly distributed in the range of 150–200 bp with a main peak of 172 bp, which met the previous criteria where cfDNA showed a specific size of ∼167 bp (Lo et al., 2010; Thierry et al., 2010). After immunoprecipitation and amplification, the size distribution profiles of all cfDNA libraries showed a range from 172 to 292 bp with a main peak of ∼292 bp including ∼120 bp sequencing adapters (Supplementary Figure 1). The cfDNA MeDIP libraries were sequenced with Illumina HiSeq 2000 (a flow chart of the steps in the analysis is presented in Figure 1). A total of 41 million raw sequenced reads were obtained from PDAC patients, 72.7% of which was mapped to the reference genome (Human hg19), and 32 million reads from healthy controls of which 54.8% was mapped. After quality filtering, there were approximately 24 million unique reads of patients and 17 million unique reads of healthy controls (Table 2).

**TABLE 1:** Clinical information of PDAC patients.

**Sample**	**Gender**	**Age**	**Stage**	**Histology**
P1	Male	59	pT3N1Mx	Ductal adenocarcinoma
P2	Male	79	pT3N1Mx	Ductal adenocarcinoma
P3	Female	67	pT2N0Mx	Ductal adenocarcinoma
P4	Female	56	pT2N0Mx	Ductal adenocarcinoma

**TABLE 2:** Statistics summary of MeDIP-seq data.

**Sample**	**Number of total reads**	**Number of mapped reads**	**Mapped read rate**	**Number of unique reads**	**Unique read rate**
P1	41,251,616	30,099,978	73.0%	25,187,519	83.7%
P2	37,618,679	27,811,589	73.9%	22,962,727	82.6%
P3	54,836,822	40,245,896	73.4%	33,248,824	82.6%
P4	31,699,187	22,318,731	70.41%	17,448,398	78.18%
C1	12,247,801	6,219,267	50.78%	5,547,597	89.20%
C2	53,490,488	31,510,659	58.91%	29,241,359	92.80%

*C, healthy control; P, PDAC patient.*

**FIGURE 2:**
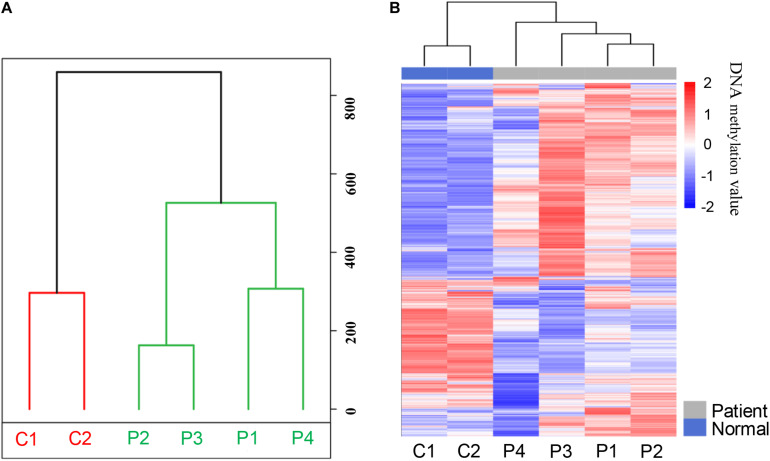
The methylation patterns of pancreatic cancer patients and healthy controls after MeDIP-seq datasets analysis. **(A)** Principal component analysis (PCA) of the methylation profiles between patients and controls. **(B)** The unsupervised cluster analysis of the genome-wide methylation profiles in patients and controls.

In order to analyze the whole-genome methylation patterns between PDAC patients and healthy controls, we performed the principal component analysis (PCA) to investigate the genome-wide methylation profiles in the two groups. The methylation patterns in PDAC patients exhibited a significant difference from the healthy control groups (Figure 2A). The unsupervised clustering analysis result further showed that there was a dramatic change in methylation patterns between PDAC patients and healthy controls (Figure 2B). This indicates that there are epigenetic differences between PDAC patients and healthy people.

### Differentially Methylated Regions of Promoters in Pancreatic Cancer Patients

**FIGURE 3:**
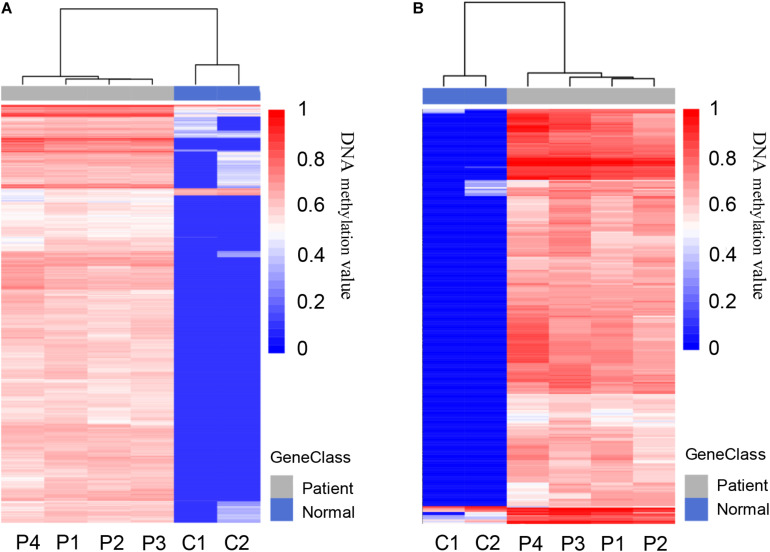
Differentially methylated regions (DMRs) in pancreatic cancer patients and healthy controls. **(A)** Heat map of total 5,205 DMRs located in the whole genome of PDAC patients compared to healthy controls, including 5,117 hypermethylated and 88 hypomethylated regions. **(B)** Heat map of total 775 DMRs located in the promoter regions of patients compared to healthy controls, including 761 hypermethylated and 14 hypomethylated regions.

A total of 5,205 differentially methylated regions (DMRs) were identified through MeDIP-seq analysis in PDAC patients (*p* < 0.05), which included 5,117 hypermethylated regions (98.3%) and 88 hypomethylated regions (1.7%) as shown in Supplementary Table 2. The clustering analysis also exhibited a significant alteration between PDAC patients and controls (Figure 3A). Previous studies have revealed that aberrant methylation patterns in the promoter region of tumor suppressor genes may cause transcriptional silencing which could be a driving force for cancer development (Herman and Baylin, 2003). We focused on promoter regions and recognized 775 different DMRs (*p* < 0.0001), including 761 hypermethylated regions (98.2%) from 532 genes and 14 hypomethylated regions (1.8%) from 14 genes (Figure 3B and Supplementary Table 3). These data suggest that most of the promoter regions are hypermethylated in pancreatic cancer samples, which is consistent with previous findings that specific hypermethylation occurring at specific promoter sites likely leads to cancer (Park et al., 2011; Liu et al., 2019; Zhang et al., 2020).

### Differentially Methylated Regions (DMRs) of CpG Regions in Pancreatic Cancer Patients

**FIGURE 4:**
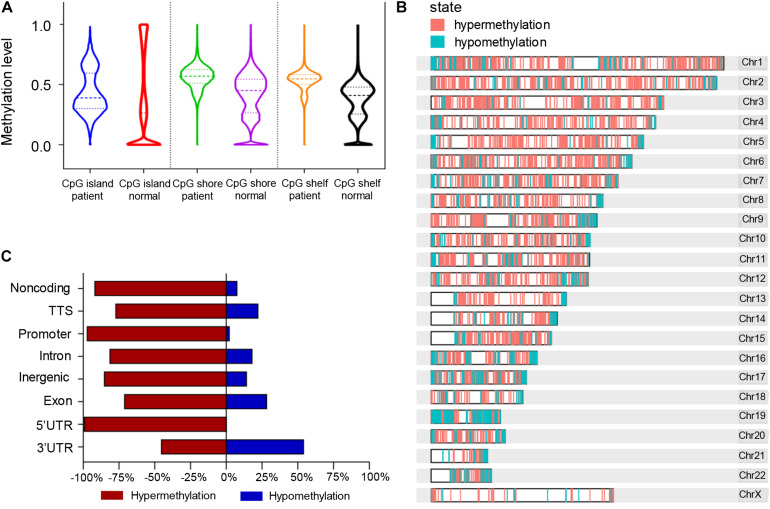
Differentially methylated regions (DMRs) of the CpG regions in pancreatic cancer patients and healthy controls. **(A)** Violin plots of DMRs located in CpG islands, CpG shores, and CpG shelfs of PDAC patients compared to controls. **(B)** Whole genomic and chromosomal location of DMRs in CGIs. **(C)** The different features of CGI distribution according to hypermethylated and hypomethylated regions.

According to the division of the CG content, some areas in the genome can be determined as CpG islands (CG content > 50%) (Gardiner-Garden and Frommer, 1987), CpG shores (up to 2 kb from CpG islands) (Irizarry et al., 2009), and CpG shelfs (≥2 kb from CpG islands) (Nones et al., 2014). It is reported that 72% of promoters are unmethylated GC-rich (Saxonov et al., 2006). Here we found that the general methylation levels of CpG regions in pancreatic cancer patients were higher than those in normal controls, which showed the median methylation levels in CGI, CpG shore, and CpG shelf to be 0.39, 0.57, and 0.5475, respectively, compared with 0.265, 0.45, and 0.41, respectively in controls (Figure 4A). Hypermethylation of CGI sites in promoter regions is considered as a risk marker for cancer development and progression (Costello et al., 2000; Esteller et al., 2001; Widschwendter and Jones, 2002), therefore, only DMR in CGIs were in focus and used for further analysis.

A total of 761 DMRs was identified in CGIs of the whole genome in PDAC patients (*p* value < 0.0001). Among them, there were 734 (96.5%) hypermethylation regions from 507 genes and 27 (3.5%) hypomethylation regions from 26 genes (Supplementary Table 4). The visual DMR signals of hypermethylation and hypomethylation in CGIs mapped to the whole genome are shown in Figure 4B. The distribution features of hypermethylated and hypomethylated regions in CGIs were further classified as shown in Figure 4C. A predominant hypermethylation of DMRs in CGIs was observed, except in the 3′UTR region (Figure 4C).

### Identification of Differentially Methylated Genes Located in Promoter CGIs in Pancreatic Cancer Patients

**FIGURE 5:**
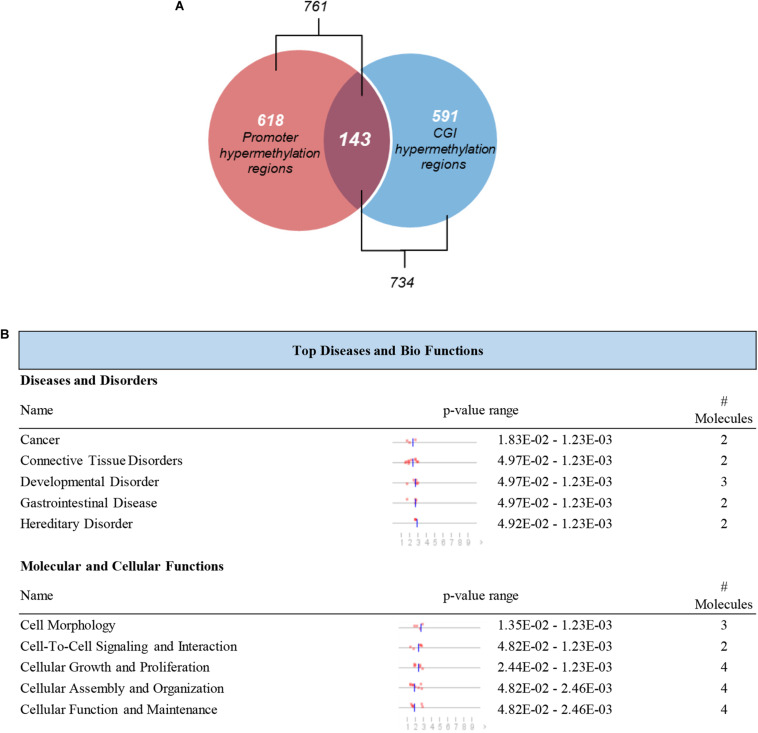
Selection and definition of differentially methylated genes in both the CGIs and promoter regions. **(A)** Hypermethylated DMRs in the overlap of promoter regions and CGIs. **(B)** Top disease and bio functions by IPA analysis for genes derived from hypermethylated DMRs located in both the promoter regions and CGIs.

It is reported that the hypermethylation of promoter CGIs is supposed to be an indicator of the risk of progression or development of cancers which is associated with the silencing of tumor suppressor genes (Feinberg, 2005; Park et al., 2011). We further screened those DMRs which were located in CGIs promoters. A total of 143 hypermethylated DMRs located in promoter regions that completely overlapped with CGIs were identified as candidate DMRs (Figure 5A). The 143 candidate DMRs were derived from 70 genes. To further understand the biological associations of the 70 genes, ingenuity pathway analysis (IPA) was performed and showed that cancer was included in the top diseases (Figure 5B).

### Cross-Validation of Potential Candidate Genes With Publicly Available DNA Methylation Data

**FIGURE 6:**
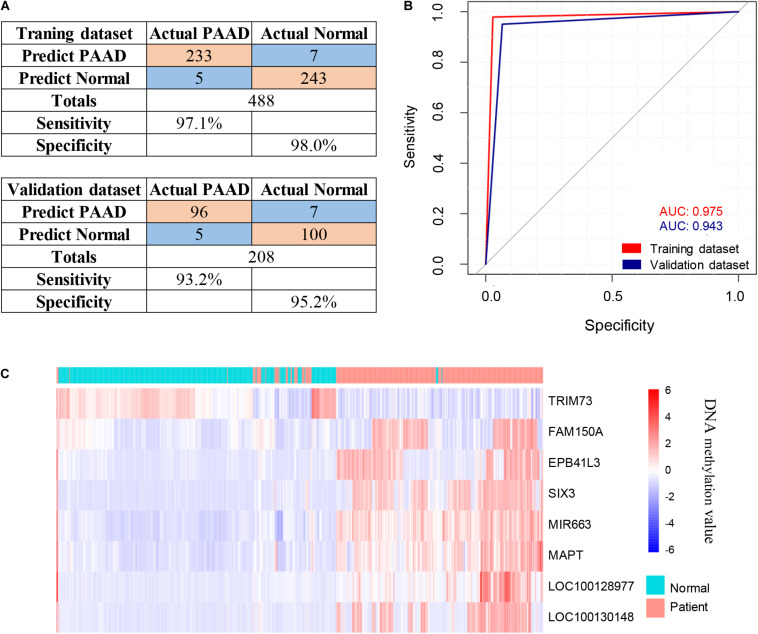
Identification of novel pancreatic cancer diagnostic markers from cfDNA methylation analysis. **(A)** Confusion tables of binary results of the diagnostic prediction model in the training and validation datasets. **(B)** ROC of the diagnostic prediction model with methylation markers in the training and validation datasets. **(C)** Unsupervised hierarchical clustering of the eight methylation markers selected for use in the diagnostic prediction model.

The 143 candidate DMRs were further annotated to 131 probes on an Illumina HM450K BeadChip Array (Supplementary Table 5) and were analyzed by the Least Absolute Shrinkage and Selection Operator (LASSO) method to select the most discriminating markers. The 75% HM450K datasets were randomly selected each time for loop modeling. Eventually, eight probes were identified as a final selection of markers which were required to appear over 450 times out of a total of 500 repetitions in the model (Table 3). To evaluate the diagnostic value of the eight markers, we built a risk prediction model in training and validation dataset using the logistic regression method. The HM450K datasets were then divided into a training cohort of 488 individuals (238 PAAD patients and 250 normal controls) and a validation cohort of 208 individuals (101 PAAD patients and 107 normal controls). The final prediction model achieved a sensitivity of 97.1% and a specificity of 98.0% on the training cohort, the sensitivity and specificity of the validating cohort was 93.2 and 95.2%, respectively (Figure 6A). This model could distinguish PAAD patients from the normal controls both in the training dataset (the area under the ROC curve, AUC = 0.975) and the validation dataset (AUC = 0.943). The prediction performance of the model in two datasets is shown in Figure 6B. To further characterize the methylation status of the eight markers in PAAD patients and normal controls, unsupervised hierarchical clustering was performed in 696 cases of the HM450K datasets (Figure 6C). The result demonstrated that these eight markers were able to distinguish PAAD patients from normal controls with high sensitivity and specificity.

**TABLE 3:** Characteristics of the eight methylation markers and their coefficients in PAAD diagnosis prediction.

**Markers**	**Ref Gene**	**Coefficients**	**SE**	***z* value**	***p*-value**
cg00394725	TRIM73	−3.1937	0.6835	−4.673	<0.05
cg09442654	FAM150A	0.3357	0.4777	0.703	<0.05
cg26170805	EPB41L3	1.8672	0.781	2.391	<0.05
cg19186145	SIX3	1.877	0.871	2.155	<0.05
cg11220245	MIR663	0.4137	0.4914	0.842	<0.05
cg11909912	MAPT	0.9288	0.5346	1.737	<0.05
cg10780632	LOC100128977	8.7616	3.4814	2.517	<0.05
cg19670923	LOC100130148	0.7289	0.8566	0.851	<0.05

*SE: standard errors of coefficients; *z* value: Wald *z*-statistic value.*

### Analysis of Relative Methylation Levels of the Eight Markers Between PAAD Patients and Normal Controls

**FIGURE 7:**
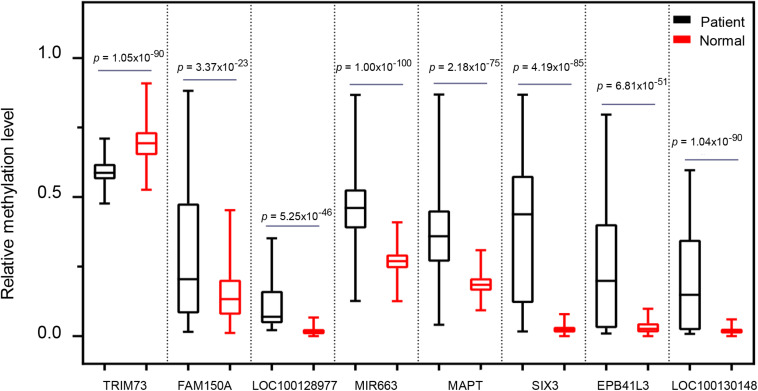
The comparison of the methylation level of the eight selected markers between pancreatic cancer patients and healthy controls.

To further address whether the eight markers we identified can distinguish pancreatic cancer patients from the healthy individuals, we next assessed the methylation levels of the eight markers in 696 cases including 339 PAAD patients and 357 normal controls. For all eight markers, there was a significantly difference in the overall methylation levels between the PAAD patients and normal controls (BH-adjusted *p* < 0.0001) (Figure 7). It suggested that the eight markers: *MAPT*, *SIX3*, *MIR663*, *EPB41L3*, *FAM150A*, *TRIM73*, *LOC100128977*, and *LOC100130148* may serve as potential biomarkers for the early diagnosis of pancreatic cancer.

## Discussion

Here, we performed a genome-wide epigenetic profiling assessment in pancreatic cancer patients for screening potential biomarkers using MeDIP-seq technology in cfDNA. Our analysis exhibited global changes in cfDNA methylation patterns in pancreatic cancer patients. In our study, we found 761 hypermethylated DMRs in promoter regions and 734 hypermethylated DMRs in CGIs derived from pancreatic cancer patients, furthermore, a total of 143 candidate DMRs were identified, located in both the promoter regions and CGIs. For subsequent analysis, tissue-derived data from TCGA and GEO was used due to the lack of cfDNA metalation data in public datasets. Finally, the diagnostic prediction model of the eight probes was established, including *MAPT*, *SIX3*, *MIR663*, *EPB41L3*, *FAM150A*, *TRIM73*, *LOC100128977*, and *LOC100130148*. Among these, *MAPT*, *LOC100128977*, and *LOC100130148* are the three differentially methylated CpG sites that hit only one gene locus. The diagnostic prediction model could effectively distinguish between PAAD patients and normal controls according to both the training cohort (AUC = 0.975) and validation cohort (AUC = 0.943). These results represented promising novel methylation markers for the early diagnosis of pancreatic cancer.

To determine the prognostic value of the eight markers in pancreatic cancer patients, Kaplan–Meier survival analysis was performed (Supplementary Figure 2). Pancreatic cancer patients with a high expression of *MAPT*, *EPB41L3*, *LOC100128977*, and *LOC100130148* had an evidently higher overall survival as compared with those with a low expression of *MAPT* (*p* = 0.0034), *EPB41L3* (*p* = 0.0088), *LOC100128977* (*p* = 0.0077), and *LOC100130148* (*p* = 0.0017). However, the multivariate Cox regression analysis indicated that *TRIM73*, *FAM150A*, *EPB41L3*, *SIX3*, *MAPT*, *LOC100128977*, and *LOC100130148* might not be independent factors for the prognosis of pancreatic cancer patients (Supplementary Table 6). This may indicate that gene expression is not only regulated by methylation, but also under a complex regulatory system. Therefore, these eight markers may be effective biomarkers for the diagnosis of pancreatic cancer, but they can not be used as prognostic indicators.

In recent years, there have been a few studies into the genome-wide detection of cfDNA methylation profiling using the MeDIP-seq method to screen potential tumor biomarkers. Shen et al. (2018) collected seven kinds of cancer samples for MeDIP-seq data analysis and took transcription factors into consideration while processing the biomarker analysis. Xu et al. (2019) identified hypermethylated DMRs in the promoter region for finding early diagnosis markers of lung cancer. In this study, we aimed to identify biomarkers in cfDNA which were located both in promoter regions and CGIs. CGIs are closely related to tumor epigenome, especially in promoter regions. Lay et al. (2015) demonstrated that compared to non-CGI promoters, methylation in CGI promoters had a greater impact on nucleosome phasing and histone modifications which have an influence on directing the functional organization of cancer epigenome. Tumorigenesis often coincides with CGI hypermethylation, leading to the inactivation of tumor suppressor genes (Namba et al., 2019). In a study of the genome-wide search for identifying potentially methylated changes during the progression of colorectal neoplasia, (Gu et al., 2019) found that hypermethylation occurred mainly in the overlap regions of CGIs and promoters, while hypomethylation tended to be far away from functional regions. Studies in hepatocellular carcinoma and ovarian cancer also revealed that the methylation status of some genes in the promoter and CGI regions can be used as prognosis markers for cancer patients (Dai et al., 2013; Lee et al., 2016).

Allele-specific methylation (ASM) has been well documented in imprinted loci. The parental allele 5^*m*^C asymmetry would create allele-specific imprinted differentially methylated regions (iDMRs). Moreover, it has been recently reported that some ASM loci undergo cancer-associated epigenetic changes in hematopoietic cancer. de Sa Machado Araujo et al. (2018) reported that the maternally inherited 5^*m*^CpG imprints for one gametic (*PARD6GAS1*) and one somatic (*GCSAML*) iDMRs are dysregulated in hematopoietic cancers. Among the eight methylated probes that could potentially serve as diagnosis markers in this study, we found four markers that were allele-specific methylated, including *EPB41L3, SIX3, MIR663, and MAPT*, suggesting that ASM also occurs in solid malignancies. Unlike whole-genome bisulfite sequencing (WGBS), which could detect the methylation state of nearly each CpG site, MeDIP technology uses an anti-methylcytosine antibody at a resolution of 100–300 bp. Therefore, MeDIP could not distinguish DNA methylation at a single base resolution (Yong et al., 2016). So ASM could not be included in the current study. Pancreatic cancer is a highly lethal disease, the lack of early detection and optional treatment is the main reason. Therefore, as a non-invasive micro diagnostic technology, cfDNA combined with MeDIP-seq is expected to be an effective method for early clinical diagnosis. In our analysis, *MAPT*, *SIX3*, *MIR663*, *EPB41L3*, *FAM150A*, *TRIM73*, *LOC100128977*, and *LOC100130148* exhibited statistically significant differences between pancreatic cancer patients and the healthy controls (Figure 7). *MAPT* is a potential predictive biomarker of the efficacy of SG410, a benzoylphenylurea sulfur analog for pancreatic cancer treatment (Jimeno et al., 2007). Tumor suppressor *SIX3* is reported to inhibit cell proliferation, migration, and invasion in glioblastoma and breast cancer (Zhang et al., 2017; Zheng et al., 2018; Yu et al., 2020). *MIR663* could act as a tumor suppressor in gastric cancer (Pan et al., 2010) and glioblastoma (Shi et al., 2014). *FAM150A* is a potential prognostic marker of clear cell renal cell carcinomas (Tian et al., 2014). Taken together, these markers, which we identified in the plasma of pancreatic cancer, may have potential clinical values.

## Conclusion

In summary, by analyzing genome-wide cfDNA methylation profiling using the MeDIP-seq method, we established a set of eight potential biomarkers which might be applied in non-invasive diagnosis of early-stage pancreatic cancer.

## Data Availability Statement

Publicly available datasets in the TCGA (https://portal.gdc.cancer.gov/projects/TCGA-PAAD) and GEO (GSE49149 and GSE40279) databases were used in this study. The pancreatic cancer patients’ raw data of MeDIP-seq in this study are available in the EMBL database (http://www.ebi.ac.uk/arrayexpress/) under accession number E-MTAB-9678, and the healthy controls C1 (ERS2672506, ERS2672505) and C2 (ERS2672508, ERS2672507) are available in the EMBL database under accession number E-MTAB-7163

## Ethics Statement

The studies involving human participants were reviewed and approved by Ethics committees of ChangHai Hospital and School of Medicine, Northwest University. The patients/participants provided their written informed consent to participate in this study.

## Author Contributions

JT and GJ conceived the study and were in charge of the overall direction and planning. JT and SyL wrote the manuscript with input from all authors. SyL and LW collected the samples. QZ and YK performed the computational framework. SyL, LW, and QZ analyzed the data. ZW and SxL provided the technical support. JT and GJ provided the funding support. All authors reviewed the manuscript and approved the final version for publication.

## Conflict of Interest

The authors declare that there is no conflict of interest regarding the publication of this paper.

## Footnotes

1 http://hgdownload.cse.ucsc.edu/goldenpath/hg19/database/cpgIslandExt.txt.gz

2 https://portal.gdc.cancer.gov/projects/TCGA-PAAD

3 https://doi.org/10.5281/zenodo.4066412

4 http://gepia.cancer-pku.cn/index.html

## Supplementary Material

Supplementary Figure 1 Representative bioanalyzer profiles of cfDNA and MeDIP-seq libraries.

Supplementary Figure 2 Kaplan–Meier analysis of PAAD patients revealed the prognosis ability of seven markers.

Supplementary Table 1 Clinical information of 696 samples from Illumina HM450K.

Supplementary Table 2 Genome DMRs identified in cfDNA of pancreatic cancer patient plasma.

Supplementary Table 3 Promoter DMRs identified in cfDNA of pancreatic cancer patient plasma.

Supplementary Table 4 CGI DMRs identified in cfDNA of pancreatic cancer patient plasma.

Supplementary Table 5 131 Illumina HM450K BeadChip Array probes corresponding to 143 candidate DMRs.

Supplementary Table 6 Multivariate Cox regression analysis shows the prognosis value of the seven markers.
