# Supplementary material for: Genome-Wide Analysis of Cell-Free DNA Methylation Profiling for the Early Diagnosis of Pancreatic Cancer
Source: Front Genet. 2020 Dec 2;11:596078. doi: 10.3389/fgene.2020.596078 (PMC7794002; doi:10.3389/fgene.2020.596078)
Supplement: Supplementary Figure 1 — Representative bioanalyzer profiles of cfDNA and MeDIP-seq libraries. [file Data_Sheet_1.PDF]

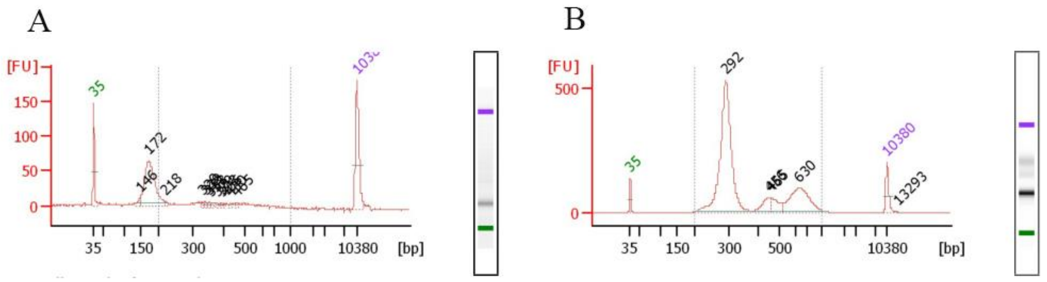

Supplementary Figure S1: Representative bioanalyzer profiles of cfDNA and MeDIP-seq libraries. (A) The fragment size distribution of cfDNA. (B) The fragment size distribution of MeDIP-seq library.
