# Supplementary material for: Genome-Wide Analysis of Cell-Free DNA Methylation Profiling for the Early Diagnosis of Pancreatic Cancer
Source: Front Genet. 2020 Dec 2;11:596078. doi: 10.3389/fgene.2020.596078 (PMC7794002; doi:10.3389/fgene.2020.596078)
Supplement: Supplementary Figure 2 — Kaplan–Meier analysis of PAAD patients revealed the prognosis ability of seven markers. [file Data_Sheet_2.PDF]

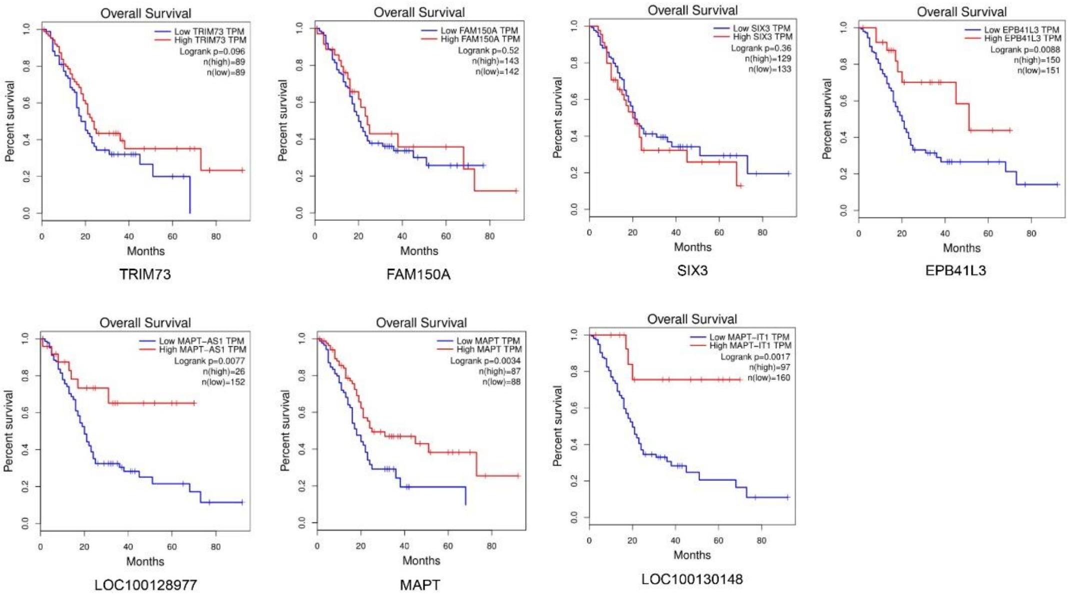

Supplementary Figure S2: Kaplan–Meier analysis of PAAD patients revealed the prognosis ability of 7 markers.
